# Oral Health Status of Patients with Mental Disorders in Southwest Ethiopia

**DOI:** 10.1371/journal.pone.0039142

**Published:** 2012-06-18

**Authors:** Biruktawit Kebede, Temam Kemal, Solomon Abera

**Affiliations:** 1 Department of Dentistry, College of Public Health and Medical Sciences, Jimma University, Jimma, Ethiopia; 2 Department of Medical Laboratory Sciences and Pathology, College of Public Health and Medical Sciences, Jimma University, Jimma, Ethiopia; 3 Department of Microbial, Cellular and Molecular Biology, College of Natural Science, Addis Ababa University, Addis Ababa, Ethiopia; Catholic University of Sacred Heart of Rome, Italy

## Abstract

**Background:**

Psychiatric disorders are known to be a risk factor for the development of different oral health problems especially for dental caries and periodontal diseases. In spite of this fact, no study has been conducted to reveal its magnitude in Ethiopia. Hence, this study was conducted to determine the oral health status of psychiatric patients at Jimma University Specialized Hospital (JUSH), Psychiatric Clinic.

**Methods:**

A hospital based cross- sectional study was used from January to May 2011. A total of 240 participants were included in the study. Dental examination was done to measure indices of oral health: decayed, missing, and filled teeth (DMFT) index and community periodontal index (CPI). Oral examination was performed using mirror, probe and explorer by experienced dental doctors. A simple random sampling technique was implemented to collect data. ANOVA test, binary logistic and multinomial logistic regression analyses were done using SPSS 16.0 statistical software.

**Results:**

The mean DMFT score among the psychiatric patients was 1.94±2.12 (mean±SD) with 1.28±1.69, 0.51±1.19 and 0.14±0.48 (mean±SD) for decayed, missed and filled teeth respectively. Only about 24% of the psychiatric patients had a healthy CPI score. Incorrect tooth brushing technique was significantly associated with a DMFT score greater than 2 (AOR = 3.58; 95% CI: 1.65, 7.79). The habit of sweet intake was also associated with dental caries (AOR = 2.91; 95% CI: 1.43, 5.95). Similarly, patients with a smoking habit also demonstrated statistically significant association with dental caries (AOR = 18.98; 95% CI: 5.06, 71.24).

**Conclusion:**

The oral health status of the psychiatric patients was poor. Thus, health education about oral hygiene should be given for psychiatric patients so they can avoid the frequent intake of sweets, smoking and learn correct tooth brushing technique.

## Introduction

Oral health is fundamental to general health and essential for wellbeing. The psychosocial impact of oral health problem significantly diminishes quality of life [1], [2]. It affects eating, talking and other social and psychological areas of life [3]. Dental caries and periodontal disease are the two most common diseases that affect oral health [4], [5]. Up to 90% of the world population could be affected with periodontal diseases demonstrating the high rates of infection. If left untreated, it can lead to progressive loss of the alveolar bone around the teeth, resulting in loosening and loss of teeth. Psychosocial factors and certain medical conditions such as diabetes and infection with human immunodeficiency virus were identified as risk factors for poor oral hygiene [6]–[8].

On the other hand, mental health is one of the fundamental components of health [9], [10]. Over 450 million people are estimated to be suffering from mental disorders in the world today [11] and it affects people at all socioeconomic levels [12]. According to a cross sectional community based study carried out in Jimma town, the prevalence of mental disorder was 22.7% [13]. Major depressive disorder and schizophrenia were the most common disorders seen in JUSH while bipolar disorder and anxiety occurred at a lesser percentage [14].

People with severe mental illness have a greater risk to oral diseases than those without. This is because of various reasons such as the type and severity of mental illness, lack of personal perception of oral health problems, poor oral hygiene, specific dental phobia, difficulty in accessing health care facilities, the side effects of psychiatric drugs, poor diet, self-neglect and dental professional’s knowledge and attitudes toward people with mental illness. At the same time dental treatment is difficult for these patients because of their lack of motivation and apathy, limited cooperation, low adaptability to new prostheses, mobility difficulties, fear of treatment, poor communication as well as financial considerations [15]–[19].

In the past two decades, it has been reported that the consequence of oral health whether from mental illness or other causes, has an effect on general health. It results in a range of medical conditions including cardiovascular diseases, type 2 diabetes, adverse pregnancy outcome, osteoporosis, aspiration pneumonia and rheumatoid arthritis. Current evidence suggests that improved oral health should be encouraged as part of the healthy lifestyle message to reduce the burden of chronic disease [20]. In Ethiopia, oral health has a low priority in the context of mental illness. To the best of our knowledge, no information is available regarding oral health among patients with mental disorders in Ethiopia. Hence, the purpose of this study was to assess oral health status of psychiatric patients at JUSH.

## Methods

### Ethical Statement

The research protocol conducted by the department of Dentistry in the Psychiatric Clinic of JUSH has been reviewed and approved by the ethical committee of Jimma University. Permission was sought from the psychiatry clinic to conduct the study. The purpose of the study was clearly explained to the participants, their guardians/care-givers and psychiatric clinic staffs. Participants fulfilling the inclusion criteria were included in the study only after obtaining an informed written consent from their guardians/care-givers as they had a reduced capacity to be consented. For literates, they themselves read the consent request. But for those who could not read, the data collectors read for them and obtained their signature or finger print to affirm their consent. To ensure participants confidentiality code numbers replaced names and no personal identifiers were included in the written questionnaires.

### Study Design and Area

A hospital based cross-sectional study was conducted in JUSH, Psychiatric Clinic. Jimma town is located south west of Addis Ababa, the capital of Ethiopia. This Psychiatric center was chosen because it is the only psychiatric clinic providing psychiatric service in South West Ethiopia for a population of 2,495,795 in the Zone.

### Participants

We included patients who had a primary diagnosis of dementia, schizophrenia, anxiety, depressive and bipolar affective disorders. These same patients whose conditions were deemed very serious thus posing limited ability to cooperate were excluded as were those with alcohol or substance use disorders, brain injury, intellectual disability and aggression tendencies.

The sample size was determined using single population proportion formula by assuming that 50% of the patients will have oral health problem to obtain maximum sample size with 95% confidence level and 5% level of significance. The total sample size was 384. From these eligible study subjects, 91(23.7%) were too ill and aggressive to continue after being engaged for the study and 53(13.8%) flatly refused to participate. As a result, the final study group included 240 (62.5%) participants.

### Measurements

Socio-demographic data was collected by a well-trained dental intern using pre-tested and structured questionnaire which had been designed based on the primary objective of the study. The questionnaire was prepared in English, translated into local languages and then re-translated back to English to check for consistency. Dental examination was carried out by two experienced dentists as required by a WHO protocol [21]. The two experienced dentists read, understood and standardized their methods of operation so as to minimize error and have reproducible data. CPI was assessed by using a North Carolina probe (HuFriedy®) calibrated every millimeter. All teeth in a sextant were recorded. Data was collected from six sites on every tooth present and registered on a chart. Mental health diagnosis was determined by mental health professionals. Our information was then obtained from medical histories after the patient diagnosis.

DMFT score and CPI were considered as dependent variables. Socio-demographic variables: age, sex, occupation, marital status, educational level; and factors predisposing for oral health problems including oral hygiene habit, frequency of tooth brushing, smoking habit, type of psychiatric disorder, duration of psychiatric illness, Khat chewing habit, sweet intake habit, medication used for the illness were taken as explanatory variables. ANOVA test, univariate and multivariate logistic regression and multinomial logistic regression analyses were done using SPSS version 16 software. The mean DMFT score of our study was 1.94±2.12, but for the purpose of comparison we rounded off the decimal and used 2 as mean DMFT.

## Results

### Study Subjects Characteristics

From a total of 240 total participants, one-hundred and sixty eight (70%) were males, psychiatric patients age ranged from15–68 (29.9±9.79; mean age (year) ±SD. One–hundred and fifty six (65.0%) were Muslim in religion, one-hundred and fifty eight (65.8%) were Oromo in ethnicity, ninety six (40.0%) had secondary school education, sixty three (26.2%) were farmers in occupation and one-hundred and fifteen (47.9%) were single in marital status category **(** **Table 1** **)**.

**Table 1:** Socio-demography of the study participants.

Socio-demographic data	Frequency (%)
**Sex**	
Male	168(70)
Female	72(30)
**Age (years)**	
15–34	164(68.3)
35–54	69(28.8)
55–68	7(2.9)
**Ethnicity**	
Oromo	158(65.8)
Amhara	58(24.2)
Tigrae	21(8.8)
Kefa	3(1.2)
**Religion**	
Muslim	156(65.0)
Orthodox	61(25.4)
Protestant	21(8.8)
Catholic	2(0.8)
**Marital status**	
Single	115(47.9)
Married	98(40.8)
Divorced	10(4.2)
Widowed	17(7.1)
**Educational status**	
Illiterate	18(7.5)
Read and write	11(4.6)
Primary school	63(26.2)
Secondary school	96(40)
Higher education	52(21.7)
**Occupation**	
Daily laborer	11(4.6)
Farmer	63(26.2)
Government employee	50(20.8)
Merchant	19(7.9)
Student	57(23.8)
Others	40(16.7)

### Mental Health

Amongst two-hundred forty study subjects, about one-hundred fifty eight (65.8%) of patients had a diagnosis of depression, forty two (17.5%) had psychotic disorders (such as schizophrenia), and twenty (8.3%) had anxiety disorder. Five (2.1%) epilepsy, four (1.7%) bipolar disorder and the remainder eleven (4.6%) had others psychiatric disorders (e.g. dementia, somatization disorder and sexual dysfunction).

The majority of patients, one hundred and fifty eight (65.8%) were receiving antidepressant (like amitriptyline, imipramine, fluoxetine). Forty-two (17.5%) and nine (3.8%) were taking antipsychotics (such as haloperidol, thioridazine, fluphenazine decanoate, chlorpromazine) and anti-convulsant drugs (like carbamazepine, phenobarbital) respectively. Only thirty one (12.9%) of subjects were taking other kind of psychiatric medications such as anxiolytics (like diazepam), mood stabilizers (like sodium valproate), and drugs for sexual dysfunction (such as sildenafil citrate).

### Oral Health Status

**Figure 1:**
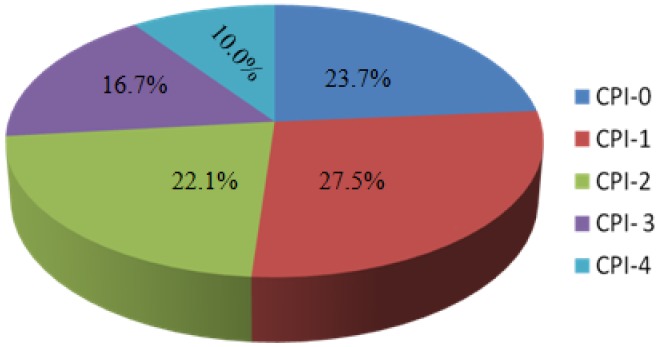
CPI score among the study participants.

The DMFT score of the study subjects ranged from 0 to 13 (1.94±2.12; mean±SD). The number of decayed teeth ranged from 0 to 11 (1.28±1.69). Most of the subjects had no filled teeth, only five (2.1%) had restorations; the number of filled teeth ranged between 0 and 4 with 0.14±0.48. The number of missing teeth ranged from 0 to 11 (0.51±1.19). The respondent’s CPI score ranged from 0 to 4 (1.6±1.28). Only about one fourth of the study subjects had a healthy CPI score of zero. Sixty six (27.5%) of the patients had bleeding and twenty four (10%) had greater than six mm pocket depth **(** **Figure 1** **)**.

### Risk Factors for Oral Health Problems

ANOVA result for the relationship between type of psychiatric illness diagnosed and DMFT (F5, 234 = 3.48, p = 0.01), filled component scores (F5, 234 = 2.52, p = 0.3), for decayed one (F5, 234 = 2.78, p = 0.02) showed statistically significant association. Whereas, missing component scores (F5, 234 = 0.89, p = 0.49) and CPI ((F5, 234 = 1.82, p = 0.11) did not.

Logistic regression analysis to determine the relationship between the dental carries status and socio-demographic characteristics is presented in Table 2. The relationship between mean DMFT score had statistically significant relation with sex and age while ethnicity, marital status, and educational level were not statistically significant (p>0.05). Males are more likely to have dental caries with DMFT greater than 2 than females [Adjusted OR = 3.23, (95% CI: 1.31, 7.98)] and as age goes up DMFT score also increases. Considering the age group 15–34 years as a reference, study subjects in the 35–54 years age group and in the 55–68 years age group were observed to have a statistically significant more likely- hood to have a DMFT score greater than 2 indicating a higher level of dental caries [AOR = 2.74; (95% CI = 1.3, 5.7) and (AOR = 20.14; (95% CI = 2.51, 161.69 respectively)] **(** **Table 2** **)**.

**Table 2:** Association of mean DMFT index and socio-demographic characteristics.

	DMFT index >2			
Variables	P –value	Crude OR(95% CI)	P –value	Adjusted OR(95% CI)
**Sex**				
Female	1	1	1	1
Male	0.001	3.79(1.76,8.16)	0.11	3.23(1.31,7.98)
**Age (years)**				
15–34	1	1	1	1
35–54	0.042	1.88(1.02, 3.47)	0.008	2.74(1.3,5.74)
55–68	0.014	8.29(1.55, 44.45)	0.005	20.14(2.51,161.69)
**Ethnicity**				
Amhara	1	1	1	1
Oromo	0.374	1.38(0.68,2.79)	0.191	1.87(0.73,4.79)
Gurage	0.126	6.92(0.58,82.55)	0.277	4.16(0.32,54.42)
Tigrae	0.168	2.13(0.73,6.24)	0.572	1.419(0.42,4.78)
**Marital status**				
Divorced	1	1	1	1
Married	0.357	2.13(0.43,10.57)	0.56	1.93(0.21,17.68)
Single	0.634	1.48(0.29,7.34)	0.66	1.66(0.17,16.13)
Widowed	0.286	0.25(0.2,3.19)	0.54	0.38(0.18, 8.3)
**Educational status**				
Illiterate	1	1	1	1
Read and write	0.99	N/A	0.999	0.0(N/A)
Primary school	0.22	2.33(0.6,8.96)	0.22	2.92(0.52,16.36)
Secondary school	0.318	1.96(0.52,7.3)	0.46	1.95(0.33,11.3)
Higher education	0.162	2.65(0.68,10.36)	0.29	2.74(0.41,18.28)

**NB:** N/A** = **Not Applicable.

Tooth brushing technique was also found to be associated with dental caries status. Psychiatric patients who were brushing their teeth incorrectly were statistically significantly more likely to have a DMFT score greater than 2 than those who brushed their teeth correctly (AOR = 3.58; 95% CI: 1.65, 7.79). Habit of sweet intake was also statistically significantly associated with dental caries. Participants with a habit of sweet intake were statistically significantly more likely to have a higher dental caries than those who had no habit of taking sweet (AOR = 2.91; 95% CI: 1.43, 5.95). Patients with smoking habit demonstrated statistical significant association with dental caries. Smokers are highly likely to have a greater DMFT score compared to non-smokers (AOR = 18.98; 95% CI: 5.06, 71.24). Likewise, type of treatment given for mental illness was also statistically significantly associated with dental caries. Psychiatric patients taking anti-depressants and anti-psychotics are statistically significantly more likely to have a bad dental hygiene (AOR = 3.73; 95% CI: 0.99, 13.93, AOR = 20.0; 95% CI: 4.66, 86.04) respectively compared to subjects with other types of treatments **(** **Table 3** **)**.

**Table 3:** Factors associated with mean DMFT index for periodontal disease.

	DMFT index >2				
Variables	Frequency (%)	P –value	Crude OR(95% CI)	P-value	Adjusted OR(95% CI)
**Tooth brushing technique**					
Incorrect	127(52.9)	0.01	2.15(1.19,3.85)	0.001	3.58(1.65,7.79)
Correct	113(47.1)	1	1	1	1
**Do you take sweet?**					
Yes	117(48.8)	0.00	2.97(1.65,5.38)	0.003	2.91(1.43,5.95)
No	123(51.2)	1			
**How often do you brush** **your tooth?**					
Once a day	17(7.1)	0.99	NA	0.99	NA
> one times a day	9(3.8)	0.99	NA	0.99	NA
Irregularly	210(87.5)	0.99	NA	0.99	NA
Don’t clean	4(1.7)	1		1	
**Do you smoke?**					
Yes	28(11.7)	0.00	2.15(1.19,3.85)	0.00	18.98(5.06,71.24)
No	212(88.3)	1		1	
**Do you chew Khat?**					
Yes	56(23.3)	0.001	8.54(3.54,20.61)	0.77	1.15(0.45,2.96)
No	184(76.7)	1		1	
**Psychiatric treatment**					
Antidepressant	158(65.8)	0.16	2.21(0.73,6.72)	0.05	3.73(0.99,13.93)
Anti-conversant	9(3.8)	0.49	2.91(0.29,12.77)	0.23	4.58(0.39,53.66)
Anti-psychosis	42(17.5)	0.001	8.17(2.43,27.49)	0.00	20.0(4.66, 86.04)
Others	31(12.9)	1		1	
**Duration of psychiatric illness (months, mean)**					
>45.9	85(35.4)	0.533	0.83(0.46,1.5)	0.057	0.46(0.21,1.02)
≤45.9	155(64.6)	1		1	

**NB:** N/A** = **Not Applicable.

For CPI score, a multiple logistic regression analysis model was employed for socio-demographic characteristics and risk factors. There was a significant association of sex, marital status and educational level with CPI **(** **Table 4** **)**. Habit of sweet intake, type of psychiatric medication, illness duration and tooth brushing technique had also significant association with CPI **(** **Table 5** **)**.

**Table 4:** Multiple logistic regression analysis model for socio-demographic characteristics against CPI score.

Variables	2 Log likelihood ofreduced model	Chi-Square	Degree of freedom	Significance
**Age**	359.41	9.36	4	0.053
**Sex**	368.26	18.21	4	0.001
**Marital status**	374.95	24.91	12	0.015
**Educational status**	385.66	35.61	16	0.003
**Ethnicity**	369.75	19.69	12	0.073

**Table 5:** Multiple logistic regression analysis model for risk factors against CPI score.

Variables	2 Log likelihood of reducedmodel	Chi- Square	Degree of freedom	Significance
**Smoking**	**351.250**	**1.912**	**4**	**0.752**
**Time of tooth cleaning**	**374.881**	**25.543**	**16**	**0.061**
**Tooth brushing technique**	**365.423**	**16.085**	**8**	**0.041**
**Chewing Khat**	**355.314**	**5.976**	**4**	**0.201**
**Habit of sweet intake**	**384.205**	**34.867**	**4**	**0.000**
**Type of medication**	**402.181**	**52.843**	**16**	**0.000**
**Illness duration**	**374.021**	**24.683**	**4**	**0.000**

## Discussion

In this study, attempts have been made to assess oral health status of psychiatric patients and factors related to dental caries and periodontal status in JUSH. The study had two limitations. First, despite its higher chance of detecting dental caries and the need for treatment, we could not use X-ray imaging; rather we used physical (dental) examination for assessing dental caries. The second was lack of literatures on the subject at a country level for comparison and discussion purposes.

Oral health status was assessed by DMFT score which is a measure of dental caries and CPI as periodontal status indicator. The mean DMFT score in our study was 1.94±2.12 (mean±SD), which was greater than general population of Ethiopian immigrants to Israel with mean DMFT of 1.48±3.16 [22]. In contrast, the findings of Jovanović *et al* [23], Zusman *et al* [16], Lewis *et al* [19], Ramon *et al* [24], Chu *et al* [25], Rekha *et al* [26] and Adeniyi *et al* [12] demonstrated mean DMFT score of 24.4, 23.8, 19.1, 17.5, 13.9, 6.1 and 2.3 respectively, which were higher than this study. The lower DMFT index in this study might be due to difference in age ranges, type of medication taken by psychiatric patients and duration of illness among study subjects. The mean number of decayed teeth in this study was 1.28. This finding was consistent with a study conducted by Kumar *et al* [27]. In contrast, it was lower than studies reported by Jovanović *et al* [23], Zusman *et al* [16] and Ramon *et al* [24]. A 0.51 mean of number of missed teeth was also reported in this study and it was lower than studies conducted in Israel [16], Serbia [23] and South Wales [19]. The mean number of filled teeth was 0.14. This finding was similar to a study conducted by Adeniyi *et al* [12] and lower than studies by Lewis *et al* [19] and Zusman *et al* [16]. This is possibly suggestive of reduced access to dental service and care of the study psychiatric patients than for those in developed countries.

Results of multiple logistic regression showed that mean DMFT was associated with sex of study subjects. Being male is a risk factor for development of dental caries. This finding is supported by the result of Jovanović *et al* [23] and male subjects might, due to culture, have a higher probability of chewing khat and smoking compared to females who generally do not indulge in these habits. At the same time, age has been associated with DMFT. An increase in age raised the mean DMFT score in the study; this was consistent with previous findings [12], [24], [27]. Marital status, ethnicity and educational level had no significant association with dental caries. Absence of association between dental caries and educational level was also reported in studies conducted by Adeniyi *et al* and Chu *et al* [12], [25].

Mean DMFT score also increased with the duration of mental illness, tooth brushing technique and sweet intake. Kumar *et al* [27] and Jovanović *et al* [23] also reported the association of DMFT scores with duration of mental illness. This study demonstrated statistical association between taking anti-psychotic and anti-depressant treatments and dental caries. The relation of anti-depressant treatment is in accordance with a study done in Serbia [23]. This could probably be because of anti-psychotropic and anti-depressant medications which result in reduced saliva thus causing mouth dryness and exposing study subjects for dental caries [26]. The association of smoking with dental caries was also observed in this study and the results are similar to studies by Millar *et al* and Ravald *et al* [28], [29]. We further found DMFT index association with chewing khat in univariate analysis. This finding may provide a new risk factor, which has not yet been reported for dental caries.

In this study, about 27% of psychiatric patients demonstrated periodontal pockets (shallow or deep pockets), whereas it was 5.3% [22] in a study conducted among the general population of Ethiopian immigrants to Israel. This clearly shows a higher degree of periodontal disease in psychiatric patients than the general population of Ethiopia.

Multinomial logistic regression analysis showed sex, marital status and educational level to be associated with periodontal status. In contrast, the finding of Jovanović *et al* [23] demonstrated its association with sex and absence of association with marital status. Females showed better periodontal conditions in this study possibly due to the culture and norm that prevents female subjects from chewing khat and smoking practices. Likewise, we found that tooth brushing technique, sweet intake habit, medication type and illness duration being statistically associated with periodontal disease predicted with CPI values. This could be explained by the fact that all of the patients already had very high CPI scores and hence there was almost no variation in this value.

In conclusion, our psychiatric patients have a poor oral health status. Therefore, health education about oral hygiene to avoid high and frequent intake of sweets, smoking and horizontal tooth brushing should be given for psychiatric patients. When psychiatric patients are prescribed for anti-depressants, the effect of the treatment should be taken into consideration and psychiatric patients could be referred to dental clinic for preventative measures.
